# Pre-existing T Cell Memory against Zika Virus

**DOI:** 10.1128/JVI.00132-21

**Published:** 2021-05-24

**Authors:** Blake Schouest, Alba Grifoni, John Pham, Jose Mateus, John Sydney, James D. Brien, Aruna D. De Silva, Angel Balmaseda, Eva Harris, Alessandro Sette, Daniela Weiskopf

**Affiliations:** a Center for Infectious Disease and Vaccine Research, La Jolla Institute for Immunology, La Jolla, California, USA; b Saint Louis University, St. Louis, Missouri, USA; c Department of Paraclinical Sciences, General Sir John Kotelawala Defense University, Ratmalana, Sri Lanka; d National Virology Laboratory, National Center for Diagnosis and Reference, Ministry of Health, Managua, Nicaragua; e Sustainable Sciences Institute, Managua, Nicaragua; f Division of Infectious Diseases and Vaccinology, School of Public Health, University of California, Berkeley, California, USA; g Department of Medicine, Division of Infectious Diseases and Global Public Health, University of California, San Diego, La Jolla, California, USA; Hudson Institute of Medical Research

**Keywords:** Zika virus, dengue virus, T cells, cross-reactivity, preexisting memory

## Abstract

The mosquito-borne Zika virus (ZIKV) has spread rapidly into regions where dengue virus (DENV) is endemic, and flavivirus cross-reactive T cell responses have been observed repeatedly in animal models and in humans. Preexisting cellular immunity to DENV is thought to contribute to protection in subsequent ZIKV infection, but the epitope targets of cross-reactive T cell responses have not been comprehensively identified. Using human blood samples from the regions of Nicaragua and Sri Lanka where DENV is endemic that were collected before the global spread of ZIKV in 2016, we employed an *in vitro* expansion strategy to map ZIKV T cell epitopes in ZIKV-unexposed, DENV-seropositive donors. We identified 93 epitopes across the ZIKV proteome, and we observed patterns of immunodominance that were dependent on antigen size and sequence identity to DENV. We confirmed the immunogenicity of these epitopes through a computational HLA binding analysis, and we showed that cross-reactive T cells specifically recognize ZIKV peptides homologous to DENV sequences. We also found that these CD4 responses were derived from the memory T cell compartment. These data have implications for understanding the dynamics of flavivirus-specific T cell immunity in areas of endemicity.

**IMPORTANCE** Multiple flaviviruses, including Zika virus (ZIKV) and the four serotypes of dengue virus (DENV), are prevalent in the same large tropical and equatorial areas, which are inhabited by hundreds of millions of people. The interplay of DENV and ZIKV infection is especially relevant, as these two viruses are endemic in largely overlapping regions, have significant sequence similarity, and share the same arthropod vector. Here, we define the targets of preexisting immunity to ZIKV in unexposed subjects in areas where dengue is endemic. We demonstrate that preexisting immunity to DENV could shape ZIKV-specific responses, and DENV-ZIKV cross-reactive T cell populations can be expanded by stimulation with ZIKV peptides. The issue of potential ZIKV and DENV cross-reactivity is of relevance for understanding patterns of natural immunity, as well as for the development of diagnostic tests and vaccines.

## INTRODUCTION

Although the incidence of Zika virus (ZIKV) infection has waned in recent years, the initial spread of ZIKV into regions where dengue virus (DENV) is endemic was associated with reports of cross-reactive immune responses among these viruses (1). The existence of some degree of cross-reactive cellular and humoral immunity is not unexpected given the phylogenetic relatedness of ZIKV and DENV. Both are members of the genus *Flavivirus* and have a high level of sequence identity at the protein level (2). The extent to which this preexisting immunity influences the clinical outcomes of heterologous infections has been demonstrated in humans and in nonhuman primates (3), and in the case of T cell immunity, evidence suggests that these responses may contribute a protective effect (4). However, the epitope targets of cross-reactive T cells have not been thoroughly characterized, thus hindering more detailed analysis of the dynamics and potential role of cross-reactive immunity in protection and immunopathology.

Previously, our laboratory showed that prior DENV exposure can shape the T cell response to subsequent ZIKV infection (5). Specifically, we found that preexisting T cell immunity in DENV-seropositive donors and recipient of the tetravalent DENV vaccine results in the cross-recognition of ZIKV peptides, and we demonstrated that DENV exposure status can affect the timing, magnitude, and protein targets of T cell responses following ZIKV infection (5). We also observed that DENV and ZIKV cross-reactive epitopes share a significantly higher level of sequence identity than the rest of the proteomes of these viruses, suggesting that these cross-reactive T cell epitopes preferentially map to conserved sites (5). T cell cross-recognition across viruses belonging to the same family has been observed in several contexts, and cross-reactivity among strains of influenza virus is a notable example (6).

More recently, we analyzed the repertoire of CD4 T cells recognizing severe acute respiratory syndrome coronavirus 2 (SARS-CoV-2) in previously unexposed individuals (7) due to the potential implications of preexisting immunity in the modulation of coronavirus disease 2019 (COVID-19) severity (8, 9). To this end, we used an *in vitro* restimulation strategy to expand populations of preexisting SARS-CoV-2-reactive memory CD4 T cells that we found to be cross-reactive with human coronaviruses (HCoV) responsible for the common cold: HCoV-OC43, HCoV-229E, HCoV-NL63, and HCoV-HKU1. This expansion of cross-reactive memory cells allowed us to map the repertoire of SARS-CoV-2 epitopes recognized in unexposed donors (7).

Here, we applied a similar strategy to define the targets of preexisting immunity to ZIKV in unexposed subjects. We analyzed samples from DENV-seropositive donors before the onset of the ZIKV epidemic to test the hypothesis that the reactive T cells would at least in part correspond to DENV-specific, cross-reactive T cells.

## RESULTS

### ZIKV-specific T cell repertoire in unexposed donors.

Previously, it was reported that DENV pre-exposure influences T cell reactivity triggered by ZIKV infection, implying that DENV infection induces ZIKV cross-reactive T cells (5). In the present study, we accordingly sought to investigate to what extent ZIKV-reactive T cell immunity exists in DENV-exposed, ZIKV-unexposed individuals. Here, we analyzed peripheral blood mononuclear cell (PBMC) samples from the regions of Sri Lanka and Nicaragua where DENV is endemic that had been obtained prior to the introduction of ZIKV in these countries. To assess the repertoire of antigen-specific CD4 T cells and detect both high and low frequencies of T cell specificities, we expanded CD4 T cells for 2 weeks in the presence of ZIKV peptide pools, as we did previously to characterize HLA-restricted CD4 responses resulting from natural DENV infection (10) and similar to the strategy we used more recently to map the repertoire of CD4 responses to SARS-CoV-2 in unexposed donors (7).

**FIG 1:**
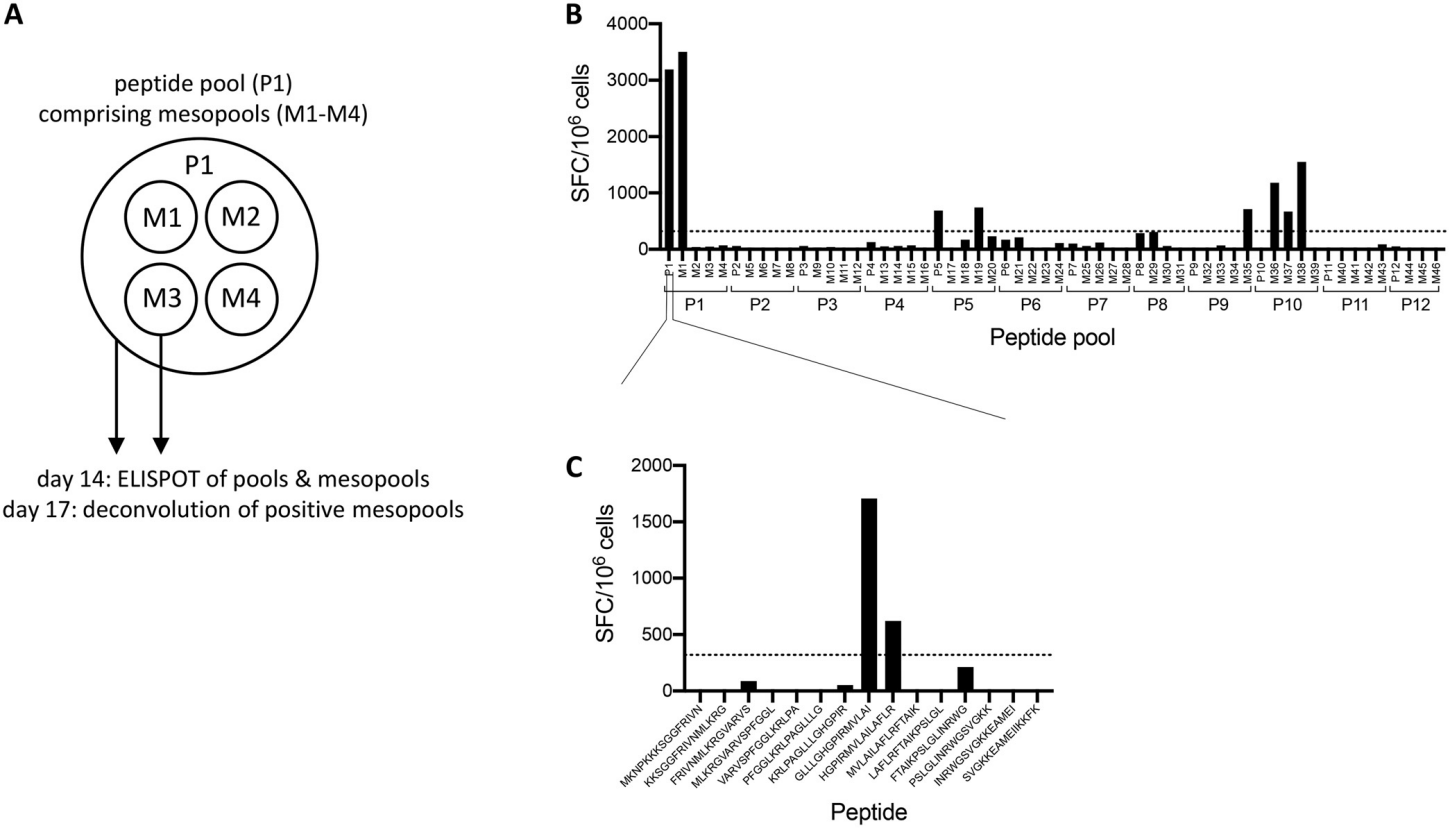
Strategy for epitope identification. Following a 14-day *in vitro* expansion, CD4 T cells were tested for reactivity to 12 pools of overlapping peptides that together spanned the ZIKV proteome. Four smaller mesopools for each of the 12 peptide pools were also tested. Three days later, positive pools were deconvoluted using individual peptides contained in the positive pool. (A) Cartoon depicting the peptide pooling strategy. (B) Number of SFCs per 10^6^ cells for one representative donor following stimulation of 14-day cultures with peptide pools and mesopools. (C) Representative deconvolution of a positive mesopool at day 17.

*In vitro* expansion was carried out by stimulating CD4 cells from each donor with 12 individual peptide pools, each averaging 60 peptides and together spanning the length of the ZIKV proteome. Following expansion, cell populations expanded from each of the 12 original pools were tested for T cell responses following stimulation with 3 or 4 smaller “mesopools” (averaging 15 peptides) for each of the original pools used for expansion. Positive responses were then deconvoluted using individual peptides contained in the positive pool to map the repertoire of ZIKV epitopes in unexposed donors (Fig. 1B and C).

**FIG 2:**
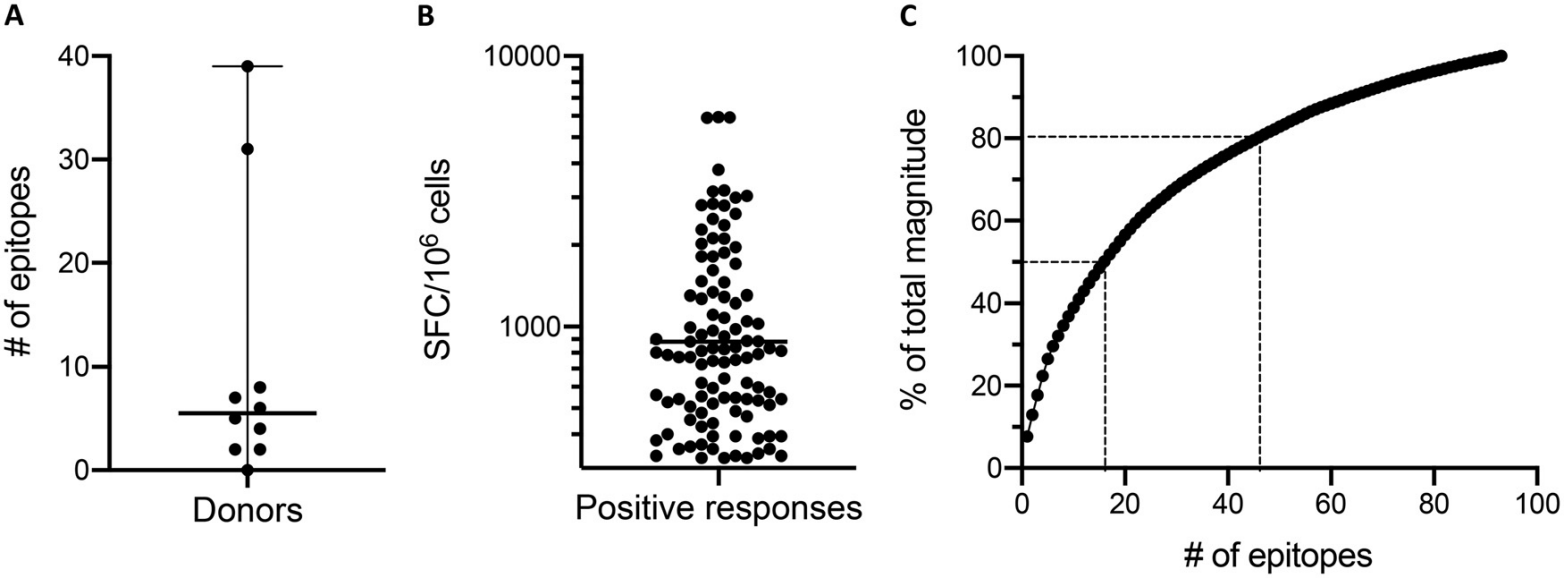
Summary of epitopes and positive responses. (A) Number of epitopes detected per donor among the 10 donors included in the study. Black lines represent the median (5.5 epitopes) and range (0 to 39 epitopes). (B) The magnitude of all positive responses detected, shown as number of SFCs per 10^6^ cells. The black line represents the geometric mean (882.1 SFCs/10^6^ cells). (C) Percentage of the total response magnitude plotted as a function of the total number of epitopes. Epitopes are ranked in descending order of magnitude, and the percentage of the total magnitude was derived from the cumulative sum. Dotted lines represent the number of epitopes associated with 50% and 80% of the total response magnitude.

In total, we identified 93 T cell epitopes, 8 of which were recognized in multiple donors (Table 1). Epitopes were identified in 9/10 donors, and the median number of epitopes recognized among the 10 donors was 5.5 (range, 0 to 39) (Fig. 2A). The average magnitude of positive responses was 882 spot-forming cells (SFCs)/10^6^ cells (Fig. 2B). In terms of coverage, the 16 most strongly recognized epitopes were associated with 50% of the total response magnitude, while 46 epitopes contributed 80% of the total magnitude (Fig. 2C).

**TABLE 1:** All epitopes identified in this study

Peptide	Peptide start site	Protein^*a*^	No. of positive donors	Frequency of responders (%)	Total no. of SFCs	Avg. no. of SFCs (positive donors)	DENV sequence identity (%)
KKSGGFRIVNMLKRG	6	ancC	1	10	833	833	53.33
FRIVNMLKRGVARVS	11	ancC	1	10	547	547	60.00
VARVSPFGGLKRLPA	21	ancC	1	10	533	533	46.67
GLLLGHGPIRMVLAI	36	ancC	2	20	2,433	1,217	66.67
HGPIRMVLAILAFLR	41	ancC	2	20	1,920	960	66.67
MVLAILAFLRFTAIK	46	ancC	1	10	980	980	60.00
LAFLRFTAIKPSLGL	51	ancC	1	10	900	900	60.00
KDLAAMLRIINARKE	86	ancC	1	10	2,020	2,020	53.33
MLRIINARKEKKRRG	91	ancC	2	20	2,267	1,133	46.67
AGEAISFPTTLGMNK	141	pr	1	10	933	933	46.67
SFPTTLGMNKCYIQI	146	pr	1	10	1,813	1,813	46.67
TKHLIRVENWIFRNP	241	M	1	10	753	753	66.67
IRCIGVSNRDFVEGM	291	E	1	10	880	880	80.00
FVEGMSGGTWVDVVL	301	E	1	10	1,467	1,467	86.67
SGGTWVDVVLEHGGC	306	E	1	10	773	773	93.33
SDMASDSRCPTQGEA	356	E	1	10	1,080	1,080	73.33
DSRCPTQGEAYLDKQ	361	E	1	10	593	593	73.33
WLVHKEWFHDIPLPW	501	E	1	10	467	467	80.00
GALEAEMDGAKGRLS	561	E	1	10	353	353	46.67
EMDGAKGRLSSGHLK	566	E	1	10	540	540	40.00
KGRLSSGHLKCRLKM	571	E	1	10	740	740	73.33
KGVSYSLCTAAFTFT	591	E	1	10	1,453	1,453	53.33
SLCTAAFTFTKIPAE	596	E	2	20	933	467	46.67
AFTFTKIPAETLHGT	601	E	1	10	367	367	60.00
TLTPVGRLITANPVI	641	E	1	10	3,153	3,153	60.00
GLNTKNGSISLMCLA	766	E	1	10	393	393	60.00
YNDVEAWRDRYKYHP	816	NS1	1	10	813	813	80.00
RMENIMWRSVEGELN	856	NS1	1	10	1,027	1,027	60.00
MWRSVEGELNAILEE	861	NS1	1	10	793	793	53.33
MWRGPQRLPVPVNEL	891	NS1	1	10	453	453	40.00
QRLPVPVNELPHGWK	896	NS1	1	10	620	620	40.00
ECPLKHRAWNSFLVE	936	NS1	1	10	507	507	66.67
SFLVEDHGFGVFHTS	946	NS1	1	10	440	440	66.67
DHGFGVFHTSVWLKV	951	NS1	3	30	2,187	729	66.67
VFHTSVWLKVREDYS	956	NS1	1	10	487	487	60.00
NDTWRLKRAHLIEMK	1001	NS1	1	10	787	787	60.00
TGGDVAHLALIAAFK	1216	NS2A	1	10	333	333	80.00
LMVLINGFALAWLAI	1271	NS2A	1	10	1,047	1,047	40.00
NGFALAWLAIRAMVV	1276	NS2A	1	10	333	333	40.00
AWLAIRAMVVPRTDN	1281	NS2A	1	10	340	340	40.00
MWHVTKGSALRSGEG	1551	NS3	1	10	540	540	60.00
ERARNIQTLPGIFKT	1606	NS3	1	10	393	393	60.00
KDGDIGAVALDYPAG	1621	NS3	1	10	1,107	1,107	60.00
ILDKCGRVIGLYGNG	1641	NS3	1	10	513	513	73.33
GRVIGLYGNGVVIKN	1646	NS3	4	40	9,607	2,402	80.00
KTRLRTVILAPTRVV	1716	NS3	2	20	6,537	3,268	86.67
PTRVVAAEMEEALRG	1726	NS3	1	10	920	920	100.00
LPVRYMTTAVNVTHS	1741	NS3	1	10	2,367	2,367	60.00
GTEIVDLMCHATFTS	1756	NS3	1	10	2,493	2,493	86.67
DLMCHATFTSRLLQP	1761	NS3	1	10	553	553	86.67
ATFTSRLLQPIRVPN	1766	NS3	1	10	327	327	80.00
PPGTRDAFPDSNSPI	1821	NS3	1	10	540	540	80.00
DHSGKTVWFVPSVRN	1856	NS3	2	20	5,120	2,560	66.67
ELMKRGDLPVWLAYQ	2036	NS3	1	10	827	827	80.00
AALKSFKEFAAGKRG	2106	NS3	1	10	353	353	73.33
FKEFAAGKRGAAFGV	2111	NS3	1	10	327	327	60.00
QEAIDNLAVLMRAET	2141	NS4A	1	10	2,113	2,113	60.00
NLAVLMRAETGSRPY	2146	NS4A	1	10	1,960	1,960	66.67
GSRPYKAAAAQLPET	2156	NS4A	1	10	883	883	53.33
GIGKMGFGMVTLGAS	2196	NS4A	1	10	833	833	53.33
IDLRPASAWAIYAAL	2301	NS4B	1	10	353	353	73.33
ASAWAIYAALTTFIT	2306	NS4B	1	10	993	993	66.67
IYAALTTFITPAVQH	2311	NS4B	1	10	803	803	53.33
MIGCYSQLTPLTLIV	2366	NS4B	1	10	380	380	60.00
ARAAQKRTAAGIMKN	2401	NS4B	1	10	5,927	5,927	86.67
KRTAAGIMKNPVVDG	2406	NS4B	1	10	5,900	5,900	93.33
NKYWNSSTATSLCNI	2481	NS4B	1	10	643	643	53.33
LAGASLIYTVTRNAG	2501	NS4B	1	10	427	427	46.67
KWKARLNQMSALEFY	2531	NS5	1	10	1,220	1,220	60.00
LNQMSALEFYSYKKS	2536	NS5	1	10	480	480	66.67
GWSYYAATIRKVQEV	2606	NS5	1	10	393	393	86.67
EEPVLVQSYGWNIVR	2631	NS5	1	10	1,613	1,613	73.33
PYTSTMMETLERLQR	2706	NS5	1	10	2,607	2,607	66.67
MMETLERLQRRYGGG	2711	NS5	1	10	387	387	60.00
KSVSTTSQLLLGRMD	2751	NS5	1	10	747	747	66.67
VTGVTGIAMTDTTPY	2856	NS5	1	10	3,793	3,793	66.67
CVYNMMGKREKKQGE	2971	NS5	1	10	520	520	93.33
GSRAIWYMWLGARFL	2991	NS5	1	10	1,267	1,267	100.00
WYMWLGARFLEFEAL	2996	NS5	1	10	1,813	1,813	100.00
GARFLEFEALGFLNE	3001	NS5	1	10	813	813	100.00
ALAIIKYTYQNKVVK	3086	NS5	1	10	327	327	80.00
VMDIISRQDQRGSGQ	3111	NS5	1	10	1,307	1,307	93.33
LNTFTNLVVQLIRNM	3131	NS5	1	10	560	560	80.00
NLVVQLIRNMEAEEV	3136	NS5	1	10	3,187	3,187	73.33
FAHALRFLNDMGKVR	3196	NS5	1	10	1,287	1,287	80.00
RFLNDMGKVRKDTQE	3201	NS5	1	10	527	527	73.33
RHQDELIGRARVSPG	3251	NS5	1	10	773	773	86.67
RETACLAKSYAQMWQ	3271	NS5	1	10	547	547	93.33
LAKSYAQMWQLLYFH	3276	NS5	1	10	2,793	2,793	86.67
RRDLRLMANAICSSV	3291	NS5	1	10	840	840	93.33
LMANAICSSVPVDWV	3296	NS5	1	10	1,340	1,340	93.33
IGDEEKYMDYLSTQV	3396	NS5	1	10	400	400	33.33
KYMDYLSTQVRYLGE	3401	NS5	1	10	2,120	2,120	40.00

a ancC, anchored capsid protein C.

**FIG 3:**
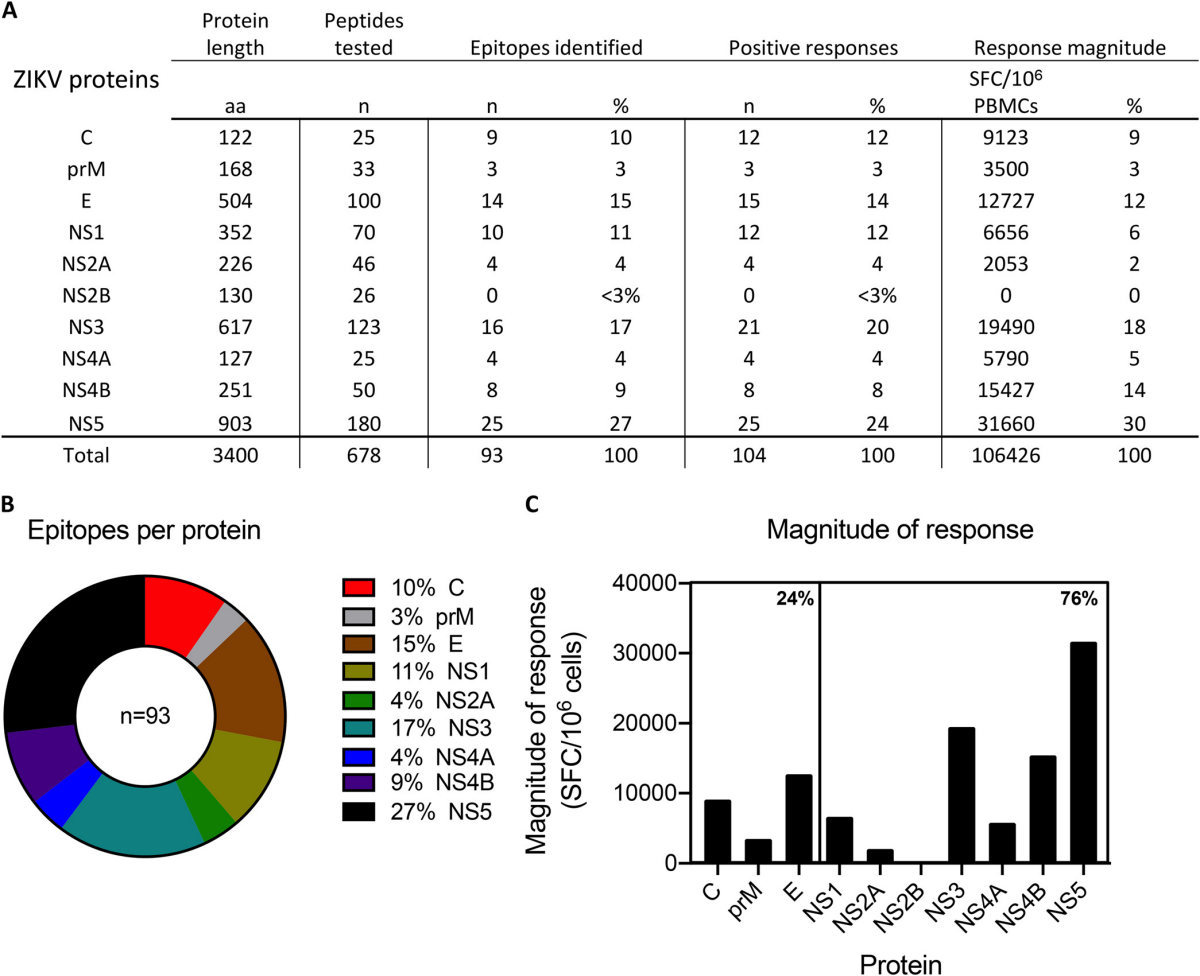
Distribution of epitopes by protein of origin. (A) Summary of epitopes, total positive responses, and response magnitude in relation to individual ZIKV proteins. These data are also presented in panel B as a parts-of-whole graph showing the number of epitopes detected per protein and in panel C as a bar graph showing the total magnitude of responses per protein. In panels A and C, the response magnitude per protein represents the sum of the total SFCs per 10^6^ cells among responding donors, and in panel C, percentages indicate the proportion of total response magnitude detected in structural and nonstructural proteins.

Positive responses were directed to every viral protein with the exception of the nonstructural protein 2B (NS2B). NS5 and NS3 were the most dominantly recognized antigens and were the sources of 25 and 16 epitopes, respectively (Fig. 3A). The next most frequently recognized antigens included envelope (E) and NS1 (Fig. 3A and B). Patterns of response magnitude (defined as the average sum of SFCs among responding donors) confirmed the dominance of NS5 and NS3, which together accounted for 48% of the total response (Fig. 3C). In sum, nonstructural proteins accounted for 76% of the total response magnitude, while 24% of the total response was directed toward structural antigens (Fig. 3C).

### Antigen size and HLA binding influence dominance of T cell responses.

**FIG 4:**
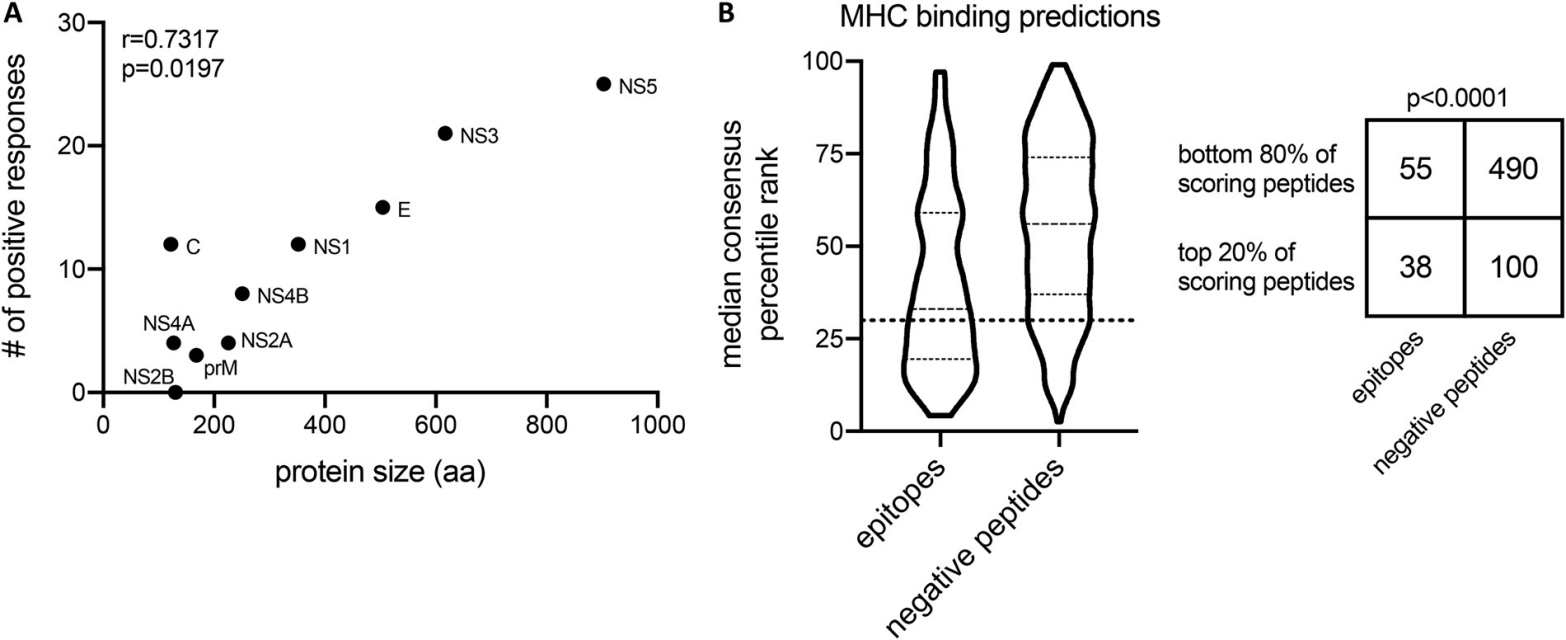
Antigen size and HLA binding determinations of T cell responses. (A) Spearman correlation of the number of positive responses detected per protein and protein size in amino acids (aa). (B) Binding predictions of peptides to class II MHC molecules using a promiscuity model. Peptides that were experimentally classified as either epitopes or negative peptides were predicted to bind a panel of 7 class II alleles, and the median consensus percentile rank of each peptide among the 7 alleles is plotted. The dotted line indicates a 20th-percentile cutoff, and peptides with a median percentile consensus rank below this cutoff represents the top 20% of predicted peptides. The number of epitopes and negative peptides above and below the 20th percentile threshold is represented in a 2 × 2 contingency table and analyzed with a Fisher’s test.

The most dominantly targeted antigens (NS3 and NS5) consisted of large viral proteins, suggesting that patterns of immunodominance could be influenced in part by protein size, reflective of the number of potential epitope candidates. To address this possibility, we plotted the number of positive responses detected per antigen as a function of protein size in amino acids. Positive responses positively correlated with protein size (*r* = 0.7317; *P* = 0.0197), suggesting that the greater number of peptides derived from larger proteins may increase the probability that epitopes are detected in those antigens (Fig. 4A).

Another factor expected to influence immunodominance at the peptide level is HLA binding, which is necessary (but not sufficient) for a peptide sequence to serve as an epitope. To address this, we scored the tested peptides using an algorithm which predicts HLA binding patterns associated with immunodominant peptides (11). This algorithm was developed based on the observation that immunodominant peptides tend to promiscuously bind multiple class II HLA molecules (11). Lower median consensus percentile rank values correspond to an increased likelihood of being an epitope, and the top 20% of peptides scored by this method usually captures 50% of the total response (11). Using this prediction strategy, we found that 38 (41%) of the peptides we identified as epitopes through enzyme-linked immunospot assay (ELISPOT) were found in the top 20% scoring peptides (Fig. 4B) (*P* < 0.0001 by Fisher’s test). Peptides with a median consensus percentile rank below the dotted line in Fig. 4B correspond to the top 20% of binding peptides. These peptides accounted for 41% (51,347 of 124,693 total SFCs) of the total response, close to the 50% of responses that this threshold was intended to capture. Overall, these results confirm that HLA binding is a variable positively associated with response magnitude.

### Analysis of positive responses as a function of DENV sequence identity.

**FIG 5:**
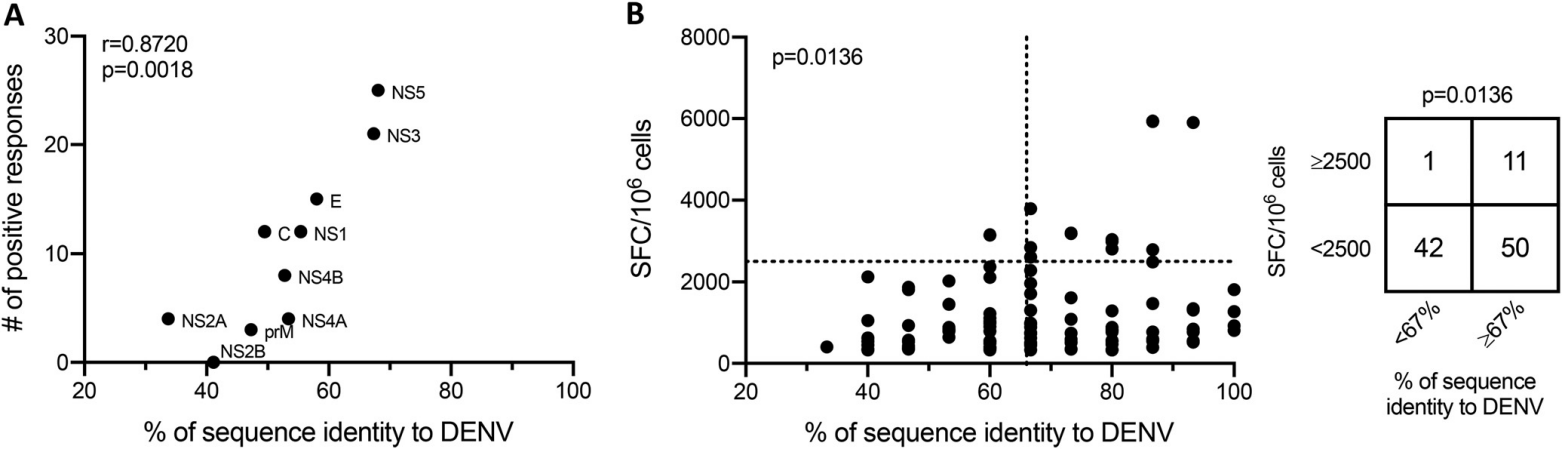
Analysis of positive responses as a function of DENV sequence identity. (A) Spearman correlation of the number of positive responses detected per protein and the sequence identity of each ZIKV protein to DENV. The maximum level of sequence identity among the 4 DENV serotypes was used for analysis. (B) Scatterplot showing the magnitude (number of SFCs per 10^6^ cells) and DENV sequence identity for all peptides that resulted in a positive response. The vertical dotted line at *x* = 66% is a threshold differentiating peptides with ≥67% and <67% sequence identity to DENV. The horizontal dotted line at *y* = 2,500 is a threshold differentiating peptides with ≥2,500 and <2,500 SFCs/10^6^ cells. A Fisher’s test was used to compare the association of sequence identity and magnitude by comparing the number of positive responses in each category.

The 2-week *in vitro* expansion assay utilized is known to detect responses originating from both naive and memory T cells (7, 10). Our hypothesis was that, at least in part, the T cell responses observed were associated with cross-reactivity originating from prior infection with one or more of the DENV serotypes. If this were to be the case, we expected that the frequency of positive responses might be tied to the level of homology between ZIKV and DENV proteins. To address this, we plotted the number of positive responses we observed at the protein level as a function of DENV sequence identity (Fig. 5A). We found a positive correlation (*r* = 0.8720; *P* = 0.0018), suggesting that the likelihood that a protein is the target of cross-reactive T cell responses is linked to the level of sequence identity of that antigen.

The finding that homology influences antigen targeting at the protein level encouraged us to explore the effects of sequence identity in greater depth. To this end, we analyzed the magnitude of positive responses as a function of sequence identity to DENV at the peptide level. Among the ZIKV peptides that were associated with the greatest response magnitude (>2,500 SFCs/10^6^ cells), we noticed that 11/12 were at least 67% identical to the homologous peptides from previously published DENV consensus sequences (Fig. 5B). We chose the 67% threshold since 1 or 2 residues in addition to a 9-mer core region are involved in the optimal binding of peptides to class II major histocompatibility complex (MHC) molecules (12, 13). Using Fisher’s test, we found that the proportion of ZIKV epitopes that resulted in a high-magnitude response was significantly different (*P* = 0.0136) between peptides with ≥67% sequence identity to DENV and those with <67% identity (Fig. 5B).

### Direct evidence of cross-reactive T cell responses.

To directly show that the most dominant epitopes identified in DENV-exposed, ZIKV-unexposed donors can be attributed to preexisting DENV-specific, ZIKV cross-reactive T cell immunity, we generated short-term T cell lines (TCLs) based on epitope/donor combinations that generated strong responses in the initial screen. We then tested the TCLs for reactivity to the homologous peptides from DENV1 to −4 as well as a panel of other flaviviruses to determine if cross-reactive T cell responses could be observed more broadly. We selected epitopes that both generated a high-magnitude response (>2,500 SFCs/10^6^ cells) and also were associated with a high level of sequence identity (>67%) to DENV, and PBMCs from the corresponding donors in which the epitopes were identified were used to generate TCLs. Notably, the NS3_1646_ epitope resulted in a high-magnitude response in multiple donors and was thus used to expand 2 TCLs. Following a 2-week expansion, we tested the TCLs for reactivity to homologous peptides from DENV1 to −4 and other flaviviruses where cell numbers permitted.

**FIG 6:**
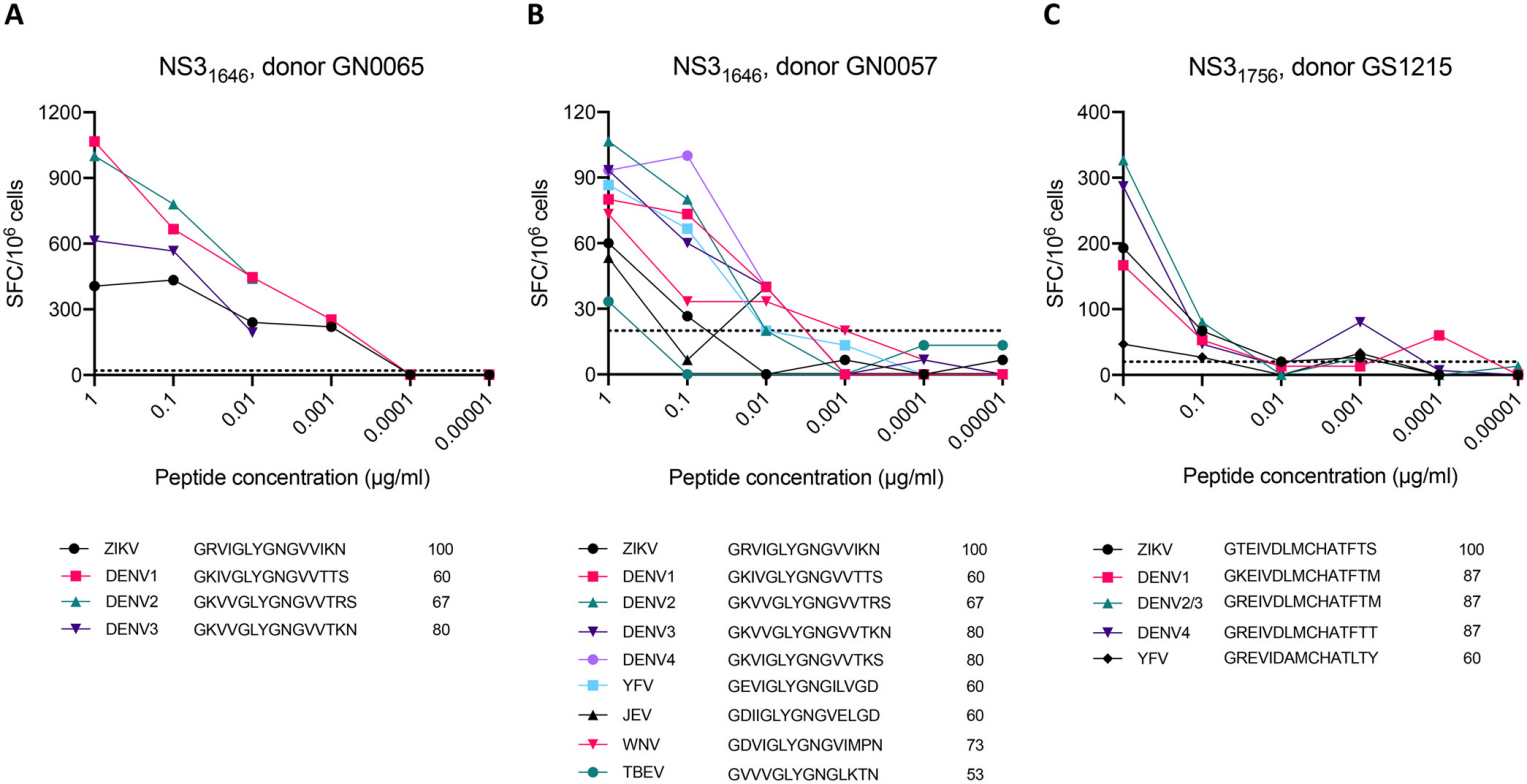
Cross-reactivity of ZIKV and homologous flavivirus peptides. Three short-term TCLs were generated based on epitope/donor combinations that generated high-magnitude responses in the primary screen. Following a 14-day *in vitro* expansion, TCLs were tested by FluoroSpot assay for reactivity to the original ZIKV peptide used for expansion as well as homologous peptides from DENV and other flaviviruses where cell numbers permitted. Keys below the graphs indicate the sequences of peptides used for analysis as well as the percent sequence identity of each peptide to ZIKV.

Among the 3 TCLs tested, all showed stronger responses to DENV homologs than to the original ZIKV peptides used for expansion (Fig. 6A to C). In TCLs from donors GN0065 and GN0057, which were both expanded using the ZIKV NS3_1646_ epitope, reactivity was greater for every DENV serotype tested (DENV1 to −3 in the case of GN0065 and DENV1 to −4 in GN0057) (Fig. 6A and B). The GN0057 TCL additionally showed stronger responses to homologs from yellow fever virus (YFV) and West Nile virus (WNV) than to the originally identified ZIKV epitope (Fig. 6B). Homologs from the more distantly related Japanese encephalitis virus (JEV) and tick-borne encephalitis virus (TBEV) also resulted in positive responses in this donor, although weaker in magnitude than ZIKV and DENV (Fig. 6B). In donor GS1215, homologs from DENV2 and −3 (the identical sequence in both viruses) and DENV4 produced stronger responses than the ZIKV NS3_1756_ peptide, while the YFV homolog, which has a lower level of sequence similarity to ZIKV NS3_1756_, resulted in a weaker response (Fig. 6C). Notably, the peptides that showed the lowest-magnitude responses among the 3 TCLs (<60 SFCs/10^6^ cells at 1 μg/ml) all had a low level of sequence identity to ZIKV (53% or 60%).

**FIG 7:**
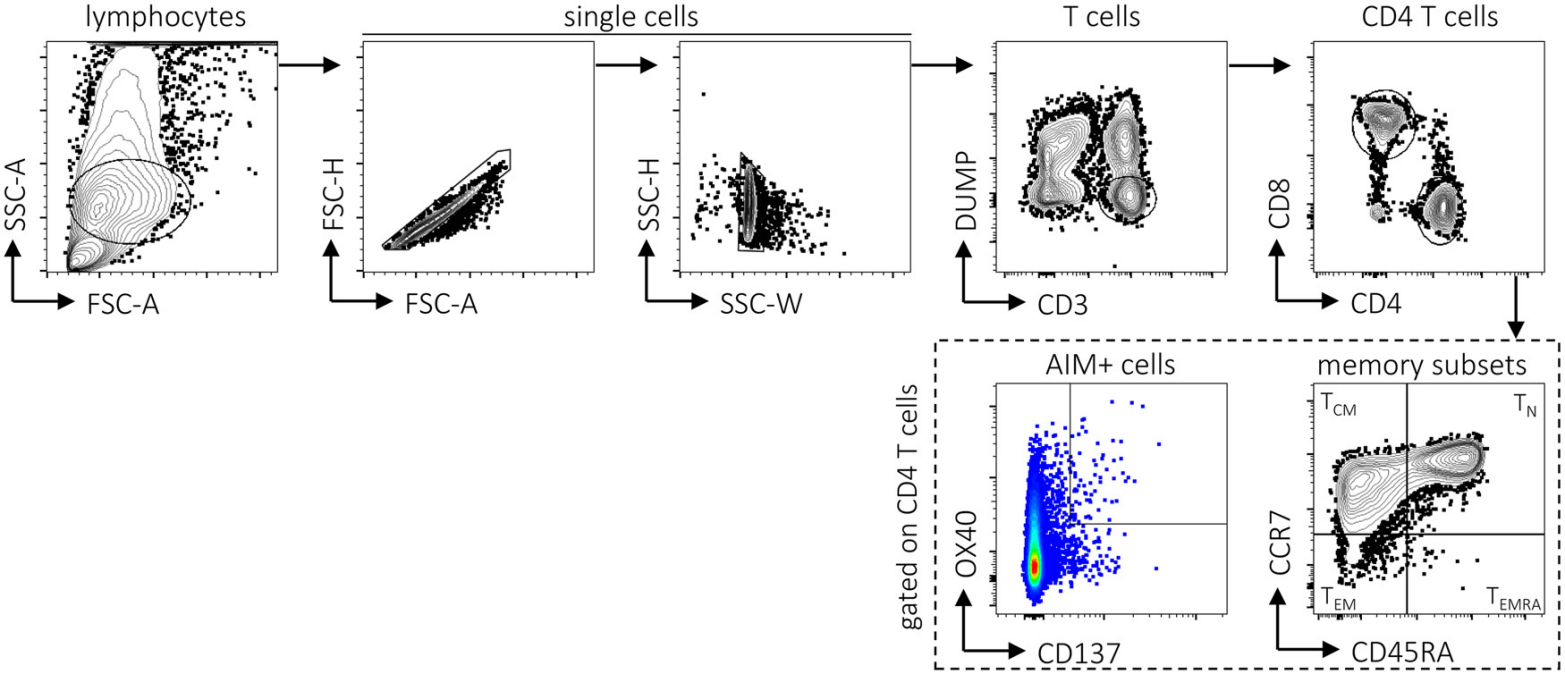
Gating strategy for AIM^+^ assay. Example of flow cytometry gating strategy for antigen-specific CD4 T cells. Antigen-specific cells were defined as double positive for the AIM markers OX40 and CD137 following *ex vivo* stimulation of PBMCs with ZIKV- and DENV-derived peptide pools. Memory phenotypes were defined based on the expression of CD45RA and CCR7 as naive (T_N_; CD45RA^+^ CCR7^+^), central memory (T_CM_; CD45RA^−^ CCR7^+^), effector memory (T_EM_; CD45RA^−^ CCR7^−^), and T_EM_ re-expressing CD45RA (T_EMRA_; CD45RA^+^ CCR7^−^).

**FIG 8:**
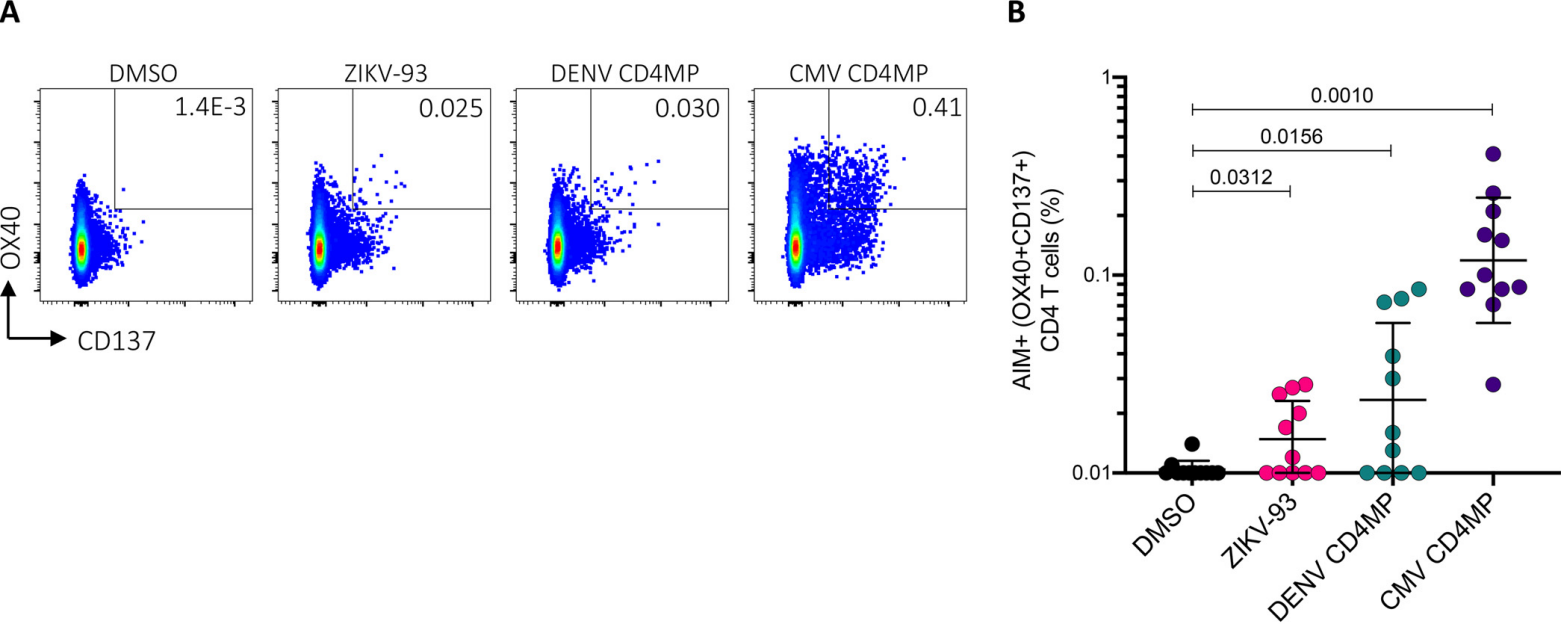
CD4 T cell responses against ZIKV epitopes *ex vivo*. (A) Example of flow cytometry gating strategy for antigen-specific CD4 T cells. The percentage of CD4 cells double-positive for the activation-induced markers (AIM^+^) OX40 and CD137 is shown. (B) Antigen-specific CD4 T cells, as measured by the percentage of cells double-positive for OX40 and CD137. PBMCs from DENV-responding individuals (*n* = 11) were stimulated overnight with the indicated peptide pools. Black lines represent the geometric mean and geometric standard deviation (SD), and pairwise comparisons were performed using a Wilcoxon test.

The 2-week *in vitro* expansion strategy utilized above was originally developed to expand low-frequency CD4 T cell responses. As further evidence of CD4 cross-reactivity between ZIKV and DENV epitopes, we performed an activation-induced marker (AIM) assay (14–16) to quantify antigen-specific cells following overnight *ex vivo* stimulation with peptide pools (Fig. 7 and 8A). For this experiment, we tested an additional set of Sri Lankan donors not used for the epitope identification studies that we previously found show CD4 T cell responses to DENV. Using a pool of 93 peptides comprising the mapped ZIKV epitopes (ZIKV-93), we measured *ex vivo* CD4 responses in several DENV-exposed individuals (Fig. 8B) (*P = *0.0312). We also observed AIM^+^ CD4 responses to the DENV CD4 megapool (Fig. 8B) (*P = *0.0156).

**FIG 9:**
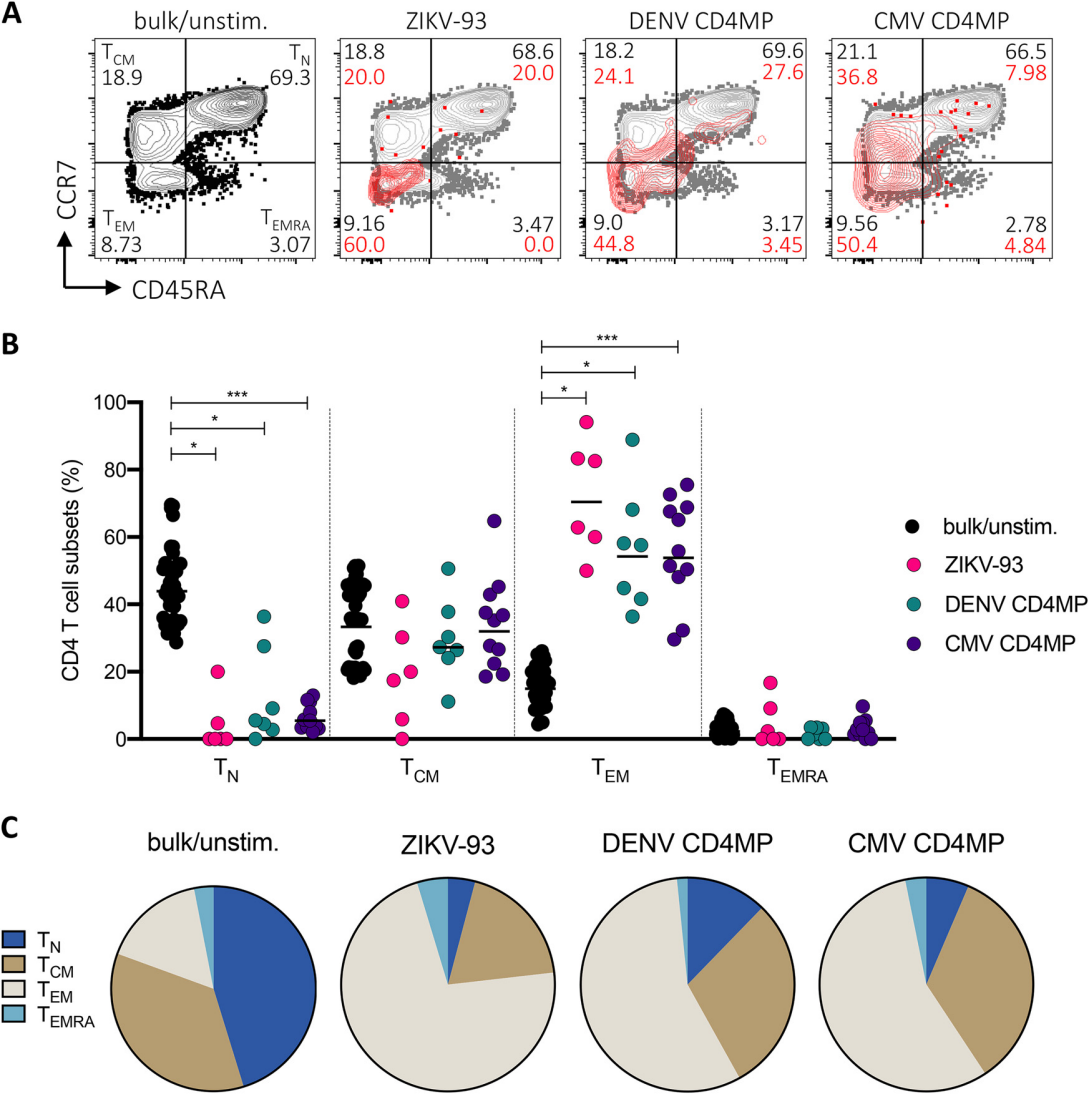
Memory phenotypes associated with CD4 T cell responses. (A) Example of flow cytometry gating strategy for memory phenotypes of AIM^+^ and bulk CD4 T cells. Percentages of bulk CD4 T cells are shown in black, and percentages of AIM^+^ cells are shown in red. (B) Memory phenotypes of antigen-specific CD4 T cells (OX40^+^ CD137^+^ T cells) following *ex vivo* stimulation with peptide pools. CD4 responses are plotted if the percentage of AIM^+^ cells was >0.01. Black lines represent geometric mean, and pairwise comparisons between AIM^+^ cells in responding donors and bulk/unstimulated populations were performed using a Wilcoxon test. (C) Average memory phenotypes associated with AIM^+^ CD4 T cell responses in DENV-exposed subjects.

Finally, to address whether the CD4 responses to ZIKV in DENV-exposed donors could be attributed to responding memory cells, we analyzed expression of the phenotypic markers CD45RA and CCR7 in AIM^+^ cells following stimulation with ZIKV and DENV peptides (Fig. 9A). We found that the majority of AIM^+^ cells showed an effector memory phenotype (T_EM_; CD45RA^−^ CCR7^−^), followed by central memory (T_CM_; CD45RA^−^ CCR7^+^) (Fig. 9B). Relative to bulk/unstimulated cells, AIM^+^ cells were significantly enriched in the T_EM_ compartment (Fig. 9B), suggesting that ZIKV-cross-reactive and DENV CD4 responses derive from memory T cells.

## DISCUSSION

Here, we investigated the repertoire of ZIKV-specific CD4 T cell responses in DENV-exposed, ZIKV-unexposed donors. The results show that in DENV-exposed donors, preexisting immunity to DENV could shape ZIKV-specific responses, and DENV-ZIKV cross-reactive T cells can be expanded by stimulation with ZIKV peptides.

In total, we identified 93 epitopes that were most abundant in nonstructural proteins, and positive responses directed toward NS3 and NS5 alone accounted for nearly half of the total response magnitude. Previously, our group mapped CD4 T cell responses to natural DENV infection in subjects from Sri Lanka (17) and Nicaragua (18) and characterized patterns of immunodominance in these populations. Notably, NS3 and NS5 were also among the most dominantly recognized antigens in these cohorts (17, 18). We also recently found that NS3 and NS5 are the most dominantly targeted YFV antigens in donors vaccinated with YF-17D (19).

In these previous studies, we also found that C was dominantly recognized by CD4 T cells (17, 18). In the present analysis, we detected relatively few C epitopes, which is consistent with the protein’s relatively small size and limited (50%) sequence identity to DENV C at the amino acid level. Our findings are similar to those of Lim et al. (20), who used a similar *in vitro* expansion method to identify cross-reactive ZIKV epitopes in DENV-immune donors. In those studies, ZIKV NS3 was consistently recognized in DENV-immune individuals, whereas ZIKV C was not (20). Overall, while both protein size and degree of DENV-ZIKV homology are correlated with the observed responses, it is noted that the correlation with degree of homology is stronger than the correlation with protein size. Intriguingly, no epitopes were identified in ZIKV NS2B, which may be reflective in part of the comparatively small size and low level of sequence identity to DENV NS2B.

In the present study, we found that high-magnitude responses were associated with peptides with ≥67% identity to the homologous DENV peptides. This 67% homology threshold was used in our previous studies of cross-reactive epitopes among common cold coronaviruses and SARS-CoV-2 (7). The results suggested that the dominant epitope responses to ZIKV sequences in DENV-exposed, ZIKV-naive donors were due to preexisting reactivity to DENV epitopes that are cross-reactive with homologous ZIKV sequences. We further showed that T cell lines specific for dominant epitopes reacted to homologous DENV peptides even more strongly than to the original ZIKV epitope used for expansion, providing direct evidence of cross-reactive T cell responses at the epitope level.

Notably, some of the strongest responses were mapped to the NS3_1646_ epitope in multiple individuals. We observed cross-reactivity of this ZIKV epitope with all flaviviruses tested. We had previously identified the same ZIKV epitope in an independent analysis of cross-reactive T cell responses among flaviviruses following attenuated yellow fever (YF-17D) vaccination (21). While cross-reactivity among YF-17D vaccinees in this previous study was generally low or absent, the NS3_1646_ epitope was widely cross-reactive among flaviviruses, including DENV1-4, ZIKV, YFV, JEV, and WNV. Moreover, the previous analysis in YF-17D vaccinees found that this epitope was restricted by the HLA-DRB1*15:01 allele (21), and here, both donors that showed a strong response to NS3_1646_ were also associated with HLA-DRB1*15:01 (data not shown).

We showed previously that T cell responses resulting from flavivirus vaccination are only marginally cross-reactive with ZIKV (21). We also previously found that DENV exposure status can positively affect the timing and magnitude of ZIKV-specific T cell responses and skew the protein targets recognized (5). Here, we reconciled these findings by characterizing the repertoire of ZIKV-specific T cell responses in DENV-immune, ZIKV-unexposed donors and showing that the most vigorous ZIKV responses are DENV cross-reactive. These results indicate that while DENV-ZIKV cross-reactivity after primary infection might be relatively infrequent (21), sequential DENV and ZIKV exposure preferentially expands cross-reactive T cell populations.

These findings can be interpreted in the broader context of ZIKV/DENV or DENV/ZIKV sequential exposure and its impact on disease severity, although the impacts of preexisting immunity in heterologous infection remain controversial (4). Our finding that DENV-exposed individuals mount T cell responses to ZIKV more quickly and with greater magnitude than DENV-seronegative donors implies that cross-reactive T cell responses could contribute to protection (5). Additionally, a study in HLA-transgenic mice identified a protective role of cross-reactive CD8 T cells in DENV-immune mice that were subsequently infected with ZIKV (22), and evidence in nonhuman primates has shown that preexisting immunity to either ZIKV or DENV does not enhance disease following infection with the heterologous virus (23–25). Studies in human cohorts from regions where DENV is endemic offer a more nuanced view of immunological cross-reactivity in sequential DENV and ZIKV infections. An analysis of a long-term Nicaraguan pediatric cohort showed that although prior DENV infection did not appear to affect the rate of total ZIKV infections, prior DENV infection did protect against symptomatic ZIKV infection (26). A study in Brazil also found prior DENV infection to be protective in relation to subsequent ZIKV (27). However, a recent analysis in the same Nicaraguan cohort showed that in the reverse series of infections, one ZIKV infection prior to DENV2 enhances the risk of severe dengue disease compared to that in flavivirus-naive subjects (28).

The immune mechanisms contributing to cross-protection are multifaceted and complex, and our findings add to a growing body of literature demonstrating cross-reactive T cell responses in infection with closely related flaviviruses. However, whether cross-reactive immune responses confer protection or predispose to severe disease is a topic of debate. The outcomes of preexisting immunity have been extensively studied in DENV, where primary infection with any of the 4 DENV serotypes affords lifelong protection to that serotype but also enhances the risk of severe disease in heterologous infection (29). On the one hand, the theory of “original antigenic sin,” which has persisted for years, postulates that the expansion of cross-reactive memory T cell populations in secondary infection dominates over the generation of new, perhaps higher-avidity T cell responses (29). While clinical data have shown that manifestations of severe dengue disease are associated with low-affinity T cell responses to the infecting virus (30) and that T cells in patients with severe disease have aberrant cytokine profiles (31), there has generally been a lack of clear evidence that original antigenic sin in relation to T cells contributes to severe disease manifestations (32).

Alternatively, it is reasonable to speculate that the selective expansion of dominant T cell responses in heterologous infections could instead favor protection from severe disease. Evidence from our group has shown that secondary DENV infection results in a honing of T cell responses to recognize conserved viral sequences, and this recognition is not associated with negative impacts on the magnitude, phenotype, or multifunctionality of CD8 T cell responses (33). Additionally, in the context of CD4 responses, we have found that donors with a history of DENV infection with different serotypes show distinct expansion of populations of terminally differentiated (T_EMRA_) cytotoxic CD4 cells, and we further showed that expansion of populations of these cells occurs in donors carrying an HLA allele associated with protection from severe disease (10). These findings, together with studies in mice that also show a protective role of T cells in heterotypic DENV infection (34, 35), suggest that cross-reactivity is a feature of the T cell response in secondary DENV infections and that preexisting immunity can hone responses and provide protection.

Beyond flaviviruses, cross-reactive T cell responses to other classes of viruses that have important implications in human health are also documented. Influenza A virus (IAV) is a notable example, since individuals are likely to undergo several infections and vaccinations over the course of a lifetime, providing ample opportunities for honing of the T cell repertoire. It has been speculated that preexisting T cell immunity confers protection to novel pandemic strains—such as A(H1N1)pdm09, which spread from Mexico to the United States in the spring of 2009—where emerging strains that share epitopes with IAV strains of previous outbreaks are less likely to be severely pathogenic (6). One study addressed this possibility using epitope mapping and detected significant recognition of A(H1N1)pdm09 epitopes in healthy donors that were conserved with seasonal H1N1 epitopes (36). Preexisting T cell immunity might account for the milder-than-expected clinical outcomes of the 2009 pandemic influenza, and it has been proposed that protection afforded through cross-reactive T cells may be a reasonable correlate of protection in more broadly protective influenza vaccines (37).

Cross-reactive T cell responses are also described in persistent viral infections such as those caused by the herpesviruses cytomegalovirus (CMV) and Epstein-Barr virus (EBV), which both induce T cell responses originating from public T cell receptors (TCRs), or TCR sequences found in multiple individuals (38). This finding has important implications in the recognition of alloantigens by cross-reactive T cell responses in transplantation (39). Finally, the implications of cross-reactive T cells in disease outcome have come under intense scrutiny recently with the detection of SARS-CoV-2 cross-reactive memory T cells in individuals not exposed to SARS-CoV-2 (7, 40–44). Preexisting immunity to SARS-CoV-2 has largely been attributed to prior exposure to the common cold coronaviruses (CCCs) HCoV-OC43, HCoV-HKU1, HCoV-229E, and HCoV-NL63, and cross-reactive T cell responses have been observed consistently in the CD4 compartment (9). Although proposed models outline a role for cross-reactive memory T cells in orchestrating a quicker antibody response, reducing lung viral loads, and promoting rapid control of the virus in the respiratory tract, the full effects of preexisting immunity on SARS-CoV-2 disease course and viral shedding remain to be determined (9).

## MATERIALS AND METHODS

### Donor cohort.

Blood donations from healthy adult donors were collected by the National Blood Center, Ministry of Health, Colombo, Sri Lanka, in an anonymous fashion between December 2010 and September 2015 and processed as previously described (17). Serology of these donors was consistent with a previous infection with at least one DENV serotype as previously described (17, 18) (data not shown). Similarly, buffy coats from blood donations were provided by the National Blood Center (NBC) of the Nicaraguan Red Cross in Managua in 2014 and processed as previously described (18). In total, PBMC samples from 6 Sri Lankan and 4 Nicaraguan donors before the global spread of ZIKV in 2016 were selected for the epitope mapping experiments. To assess *ex vivo* CD4 T cell responses to ZIKV epitopes, we used PBMC samples from an additional set of Sri Lankan donors (*n* = 11) known to show CD4 responses to DENV. These donors were independent from those used for the epitope mapping experiments.

### Peptide pooling strategy.

Peptide selection was based on the BeH818995 ZIKV isolate (accession no. AMA12084.1), from which we synthesized a panel of 15-mer peptides overlapping by 10 residues. Together, 683 peptides that spanned the length of the ZIKV proteome were synthesized, resuspended in dimethyl sulfoxide (DMSO), and divided into 12 pools, each containing an average of 60 peptides. We designed smaller peptide pools with approximately 15 peptides each and called them mesopools, and these were designed such that each of the 12 larger pools described above was composed of 3 or 4 mesopools (Fig. 1A).

### *In vitro* expansion of CD4 T cell populations and IFN-γ ELISPOT assay.

CD4 T cells were purified from PBMC samples by negative selection using the CD4 T cell isolation kit (catalog no. 130-091-155; Miltenyi Biotec) according to the manufacturer’s instructions. CD4 cells were cultured at 37°C and 5% CO_2_ in RPMI 1640 supplemented with 5% human serum at a density of 2 × 10^6^ cells per well in 24-well plates. Autologous antigen-presenting cells (APCs) were added at a ratio of 1:2 (i.e., 1 × 10^6^ APCs and 2 × 10^6^ CD4 cells), and cells were stimulated individually with the 12 ZIKV peptide pools (1 μg/ml) described above. Interleukin 2 (IL-2; 10 U/ml) was added every 3 to 4 days until cell harvest at day 14 (for peptide pool evaluation) or day 17 (for individual peptide evaluation).

Following *in vitro* expansion at day 14, ZIKV-specific responses were assessed by gamma interferon (IFN-γ) ELISPOT using the peptide pools used for *in vitro* expansion as well as the smaller mesopools. At day 17, positive pools were deconvoluted by ELISPOT using individual ZIKV peptides (10 μg/ml) contained in the positive pool. Briefly, polyvinylidene fluoride (PVDF) plates (Millipore) were coated with anti-human IFN-γ (1-D1K; Mabtech), and cells were plated in triplicate at 5 × 10^4^ cells per well. Cells were stimulated with either peptide pools (1 μg/ml) or individual peptides (10 μg/ml) in 0.1 ml complete RPMI and incubated for 20 h at 37°C and 5% CO_2_. Following stimulation, cells were discarded, and plates were incubated with biotinylated IFN-γ monoclonal antibodies (MAb) (7-B6-1; Mabtech) for 2 h at 37°C and developed as described previously (33). Positive responses were identified as those having >320 spot-forming cells (SFCs) per million cells, a stimulation index of >2, and a *P* value of <0.05 when compared to unstimulated cells using a *t* test as previously described (10).

### HLA binding predictions.

HLA binding analysis was carried out using the Immune Epitope Database and Analysis Resource (IEDB) (45). ZIKV peptides were broadly assessed for their binding to HLA class II molecules using a 7-allele promiscuous method with a 20% cutoff. This method was previously developed based on the observation that a set of 7 DRB1 and DRB3/4/5 alleles covering the main class II supertypes can be used to utilize the promiscuous binding capacity of peptides to predict epitopes in the general worldwide population (11). Calculations were performed for the 683 ZIKV peptides included in the present study using the IEDB-recommended 2.22 method, which employs a consensus method (46) comprising the combinatorial library (47), SMM-align (48), and NN-align (49) algorithms, followed by NetMHCIIpan3.2 (50). Percentile rank values were extracted, and the median consensus percentile rank was calculated for each peptide. A percentile rank threshold of 20% was used to compare the number of top-scoring peptides among experimentally identified epitopes and negative peptides.

### Sequence identity calculations.

To assess the level of homology between ZIKV- and DENV-derived peptides while accounting for differences among DENV serotypes, we calculated the sequence identity of each ZIKV peptide to consensus sequences for DENV1 to −4 using the ImmunomeBrowser tool (51) as described previously (21). Briefly, we calculated the sequence identity of each ZIKV peptide to previously published consensus sequences for DENV1 to −4, which were generated based on thousands of sequences from geographically disparate regions (52), and the maximum level of sequence identity for each peptide among the DENV serotypes was analyzed.

Similarly, to calculate the level of sequence identity between whole proteins while accounting for sequence diversity among DENV1 to −4, the coding sequences of ZIKV (AMA12084.1) proteins were compared to previously published consensus sequences for DENV1 to −4 (52). The NCBI protein BLAST algorithm (https://blast.ncbi.nlm.nih.gov) was used with default parameters for these calculations (expect value threshold = 0.05; scoring parameter = BLOSUM62 matrix; gap cost existence = 11; gap cost extension = 1; compositional adjustments = conditional compositional score matrix adjustment). As in the peptide-level analysis, the maximum level of sequence identity among the DENV1 to −4 consensus sequences was retained for analysis.

### *In vitro* expansion of T cell lines for peptide evaluation.

To characterize the cross-reactivity of T cell responses among flaviviruses in greater detail, we established short-term T cell lines (TCLs) based on epitope/donor combinations that generated high-magnitude (SFCs/10^6^ cells ≥ 2,500) responses and occurred in peptides with high homology to DENV (≥67%) in the initial screen. This resulted in the expansion of 3 TCLs. To generate each cell line, 12 × 10^6^ PBMCs were plated at a density of 4 × 10^6^ cells/ml in 6-well plates in the presence of individual ZIKV peptides (1 μg/ml) for 14 days in complete RPMI at 37°C and 5% CO_2_, with IL-2 (10 U/ml) added at days 4, 7, and 11. A panel of flavivirus peptides homologous to the originally identified ZIKV epitopes were synthesized based on reference flavivirus sequences, including DENV1 to −4 (accession no. NP_059433.1, ADK37484.1, ACW82883.1, and AIG60035.1), yellow fever virus (YFV; Q6DV88.1), Japanese encephalitis virus (JEV; AAD20233.1), West Nile virus (WNV; YP_001527877.1), and tick-borne encephalitis virus (TBEV; ADX07734.1). Following expansion, TCLs were tested for cross-reactivity to flavivirus peptides using an IFN-γ/IL-5 dual-color FluoroSpot assay as described previously (7). Each peptide was tested at 6 concentrations beginning at 1 μg/ml, with 10-fold serial dilutions down to 0.00001 μg/ml, where cell numbers permitted.

### AIM assay.

Activation-induced marker (AIM) assays were performed as previously described (7). Briefly, PBMCs were cultured at 2 × 10^6^ cells per well in 96-well U-bottom plates for 24 h in the presence of the peptide pool (1 μg/ml), an equimolar volume of DMSO as a negative control, or phytohemagglutinin (PHA; 5 μg/ml) (Roche) as a positive control. Pools used for stimulation included a cytomegalovirus (CMV) CD4 megapool used here as an additional positive control, ZIKV-93 (which consists of the ZIKV epitopes identified by ELISPOT), and a DENV CD4 megapool, a reagent our lab developed to capture CD4 responses to DENV in the general population (18). Following stimulation, cells were stained with the antibody panel shown in Table 2 and acquired on a ZE5 flow cytometer (Bio-Rad). Data were analyzed using FlowJo v10. Donors were excluded from the analysis if the frequency of CD3^+^ cells was <20% (of single cells), if the frequency of CD4 T cells was <20% (of CD3^+^ cells), or if there was a high level of background in DMSO-only-treated cells (>0.02%). For memory phenotyping, responses that exceeded a positivity rate of 0.01% were included in the analysis.

**TABLE 2:** AIM assay antibody panel

Antibody	Fluorochrome	Clone	Vendor	Catalog no.	Dilution
CD45RA	BV421	HI100	BioLegend	304130	1:50
CD14	V500	M5E2	BD	561391	1:50
CD19	V500	HIB19	BD	561121	1:50
Live/Dead	ef506/Aqua	Thermo Fisher	65-0866-18	1:200
CD4	BV605	RPA-T4	BD	562658	1:25
CD8	BV650	RPA-T8	BioLegend	301042	1:25
CCR7	FITC	G043H7	BioLegend	353216	1:50
CD69	PE	FN50	BD	555531	1:10
OX40	PE-Cy7	Ber-ACT35	BioLegend	350012	1:50
CD137	APC	4B4-1	BioLegend	309810	1:50
CD3	AF700	UCHT1	Thermo Fisher	56-0038-42	1:75

### Statistical analysis.

Statistical analysis was performed using GraphPad Prism v8.4, and details of the statistical tests used in individual analyses are included in their respective figure legends. Briefly, a Spearman correlation was carried out to assess the antigen size and DENV sequence identity determinations of positive responses, and Fisher’s test was used to analyze MHC binding predictions and the impact of DENV sequence identity on response magnitude at the peptide level. A Wilcoxon test was used to analyze AIM^+^ CD4 responses as well as the memory phenotypes associated with these responses.
